# Antifungal Activity of Cell-Free Filtrate of Probiotic Bacteria *Lactobacillus rhamnosus* ATCC-7469 against Fungal Strains Isolated from a Historical Manuscript

**DOI:** 10.3390/microorganisms11051104

**Published:** 2023-04-23

**Authors:** Mahmoud Abdel-Nasser, Gomaa Abdel-Maksoud, Ahmed M. Eid, Saad El-Din Hassan, Aya Abdel-Nasser, Maha Alharbi, Amr Elkelish, Amr Fouda

**Affiliations:** 1 Department of Manuscripts Conservation, Al-Azhar Al-Sharif Library, Cairo 11511, Egypt; 2 Conservation Department, Faculty of Archaeology, Cairo University, Giza 12613, Egypt; 3 Department of Botany and Microbiology, Faculty of Science, Al-Azhar University, Cairo 11884, Egyptsaad_hassan@azhar.edu.eg (S.E.-D.H.); 4 Food Toxicology and Contaminants Department, National Research Centre, Giza 12622, Egypt; 5 Department of Biology, College of Science, Princess Nourah Bint Abdulrahman University, P.O. Box 84428, Riyadh 11671, Saudi Arabia; maalharbi@pnu.edu.sa; 6 Biology Department, College of Science, Imam Mohammad Ibn Saud Islamic University (IMSIU), P.O. Box 90950, Riyadh 11623, Saudi Arabia; 7 Botany and Microbiology Department, Faculty of Science, Suez Canal University, Ismailia 41522, Egypt

**Keywords:** probiotic bacteria, *Lactobacillus* spp., biocontrol, historical manuscript, fungi, analytical techniques

## Abstract

Herein, twelve fungal strains were isolated from a deteriorated historical manuscript dated back to the 18th century. The obtained fungal strains were identified, using the traditional method and ITS sequence analysis, as *Cladosporium herbarum* (two strains), *Aspergillus fumigatus* (five strains), *A. ustus* (one strain), *A. flavus* (two strains), *A. niger* (one strain), and *Penicillium chrysogenum* (one strain). The ability of these fungal strains to degrade the main components of the paper was investigated by their activity to secrete extracellular enzymes including cellulase, amylase, gelatinase, and pectinase. The cell-free filtrate (CFF) ability of the probiotic bacterial strain *Lactobacillus rhamnosus* ATCC-7469 to inhibit fungal growth was investigated. The metabolic profile of CFF was detected by GC-MS analysis, which confirmed the low and high molecular weight of various active chemical compounds. The safe dose to be used for the biocontrol of fungal growth was selected by investigating the biocompatibility of CFF and two normal cell lines, Wi38 (normal lung tissue) and HFB4 (normal human skin melanocyte). Data showed that the CFF has a cytotoxic effect against the two normal cell lines at high concentrations, with IC_50_ values of 525.2 ± 9.8 and 329.1 ± 4.2 µg mL^−1^ for Wi38 and HFB4, respectively. The antifungal activity showed that the CFF has promising activity against all fungal strains in a concentration-dependent manner. The highest antifungal activity (100%) was recorded for a concentration of 300 µg mL^−1^ with a zone of inhibition (ZOI) in the ranges of 21.3 ± 0.6 to 17.7 ± 0.5 mm. At a concentration of 100 µg mL^−1^, the activity of CFF remained effective against all fungal strains (100%), but its effectiveness decreased to only inhibit the growth of eight strains (66%) out of the total at 50 µg mL^−1^. In general, probiotic bacterial strains containing CFF are safe and can be considered as a potential option for inhibiting the growth of various fungal strains. It is recommended that they be used in the preservation of degraded historical papers.

## 1. Introduction

The term “probiotic” has been highly regarded since the 1960s. Initially, it referred to chemical compounds secreted by microbes that have beneficial effects on the human body. Then, in 1980, a few distinct traits were added to this description. Recently, probiotics were described as “strains that have a favorable impact, non-toxic, non-allergic, and nonpathogenic, available in vast amounts as live cells, compatible for the environment of the gut, and storable as well as stable” [1].

Nowadays, probiotic strains of *Lactobacillus* and *Bifidobacterium* are very popular because of their many antibacterial and antifungal activities. Probiotics are bacteria that have numerous advantages for the host, most notably protection against pathogen invasion and improved nutritional viability. In addition, probiotic strains provide protection against pathogens that can infect people and cause illness. The most commonly found probiotic strains include lactic acid bacteria, as well as some types of yeast and mold, which are also used as biocontrol agents [2].

Microorganisms, especially fungi, pose a threat to the health of restorers, museum employees, and visitors in museums, libraries, and archives due to their potential to induce allergies and their generation of mycotoxins, as well as their capacity to infect people systemically. In museum storage areas, airborne fungal spore concentrations can easily exceed 8000/m^3^, including fungal diseases such as *Aspergillus niger*, *A. flavus*, *A. terreus*, *A. ustus*, *A. alliaceus*, *A. udagawae*, *A. quadrilineatus*, *A. lentulus*, and *Stachybotrys chartarum* [3,4].

On the other hand, fungi are among the most harmful species that contribute to the biodegradation of historical organic materials including books, manuscripts, and book bindings made of vegetable-tanned leather [5]. The penetration of fibers by fungal hyphae can cause mechanical stress on the components of the paper, weakening them, and they secrete extracellular enzymes, leading to biodeterioration [6,7]. The identification of different fungal strains associated with historical papers is the critical step to exploring how they colonize and degrade substrates because only a few organisms can utilize the majority of the substrates [5]. According to the published literature, the most commonly found fungal strains associated with paper damage and biodeterioration were *Aspergillus niger*, *A. flavus*, *A. terreus*, *A. ochraceous*, *A. carbonarius*, *A. fischeri*, *A. fumigatus*, *A. tamarii*, *Eurotium chevalieri*, *Cladosporium cladosporioides*, *Fusarium poae*, *Wallemia sebi*, *Penicillium notatum*, *P. oxalicum*, *P. rubrum*, and *Aletrnaria alternata* [8,9]. Researchers have used various treatment protocols to control fungal deterioration, such as chemical compounds, volatile oils, ozones, etc. The current methods used to treat paper deterioration have negative impacts on paper fibers, ink, and workers. Moreover, they are not environmentally friendly. Therefore, it is essential to discover new active substances that are safe, fast, cost effective, and ecologically friendly, in order to address this problem.

Hence, the main goal of this study is to investigate the effectiveness of using the probiotic strain, *Lactobacillus rhamnosus* ATCC-7469, as a safe, environmentally friendly, quick, and cost-effective method for controlling severely damaged fungal strains obtained from historical manuscripts. To the best of our knowledge, this study represents the first attempt to utilize probiotic metabolites which could potentially be used in the preservation field to control deteriorated fungal strains isolated from historical papers.

To explore this hypothesis, a highly deteriorated historical manuscript was selected as a source of fungal isolation. To determine the extent of biodeterioration, a range of methods, including photographic documentation, environmental scanning electron microscopy (ESEM), and X-ray diffraction (XRD) were employed. Traditional methods were used to identify various fungal strains associated with deteriorated samples, and these identifications were confirmed with molecular techniques, specifically ITS sequence analysis. Enzymatic activity was explored to determine the role of the isolated fungal strains in biodeterioration. Finally, metabolites of the probiotic bacterial strain *L. rhamnosus* ATCC 7469 were utilized to biocontrol the isolated fungal strains, using a safe dose that was determined by testing the biocompatibility of the metabolites on two normal cell lines (normal skin and lung cell lines). The metabolic profile of the probiotic bacterial strain was analyzed using gas chromatography–mass spectrometry (GC-MS).

## 2. Materials and Methods

### 2.1. Materials

#### 2.1.1. Historical Manuscript Studied

The historical manuscript named “*Explanation ‘abi Al-Muntaha on Al-fiqh Al-akbar of Imam Abu Hanifa*” dated to 1153 AH, 1741 AD, and preserved in Al-Azhar Library, Cairo, Egypt, was selected for the isolation of fungal strains due to the presence of high deterioration. This historical manuscript was written in Arabic using carbon ink and has a size (cm^3^) of 22 × 16 × 2 (length × width × height). The preservation conditions were as follows: temperature, 27 °C; relative humidity, 60%; and the manuscript was kept in tightly closed iron cabinets on iron shelves, and there was no air conditioning system present. As a result, the preservation area has poor ventilation.

#### 2.1.2. New Whatman Paper (Control)

Whatman paper (No. 1) was used as a control in all analyses and investigations used to compare with the historical paper manuscript studied. The Whatman paper was purchased from El-Gamhorya Company, Cairo, Egypt.

### 2.2. Methods

#### 2.2.1. Measurement of Deterioration Aspects

##### Visual Assessment

The manuscript was assessed visually using the critical eye of the conservator and a digital camera (Samsung camera 38MP, f/2.2 lens slot) to record the aspects of deterioration found on the components of the studied manuscript.

##### Environmental Scanning Electron Microscope (ESEM) Investigation

Whatman paper No. 1 (control for papers) and vegetable-tanned (mimosa) goatskin (control for leather) were used to investigate the surface morphology and deterioration aspects of the studied samples and to identify the fibers used in the paper sheets and bookbinding of the studied manuscript [10]. The investigation was conducted using a Quanta 3D 200i (Boynton Beach, FL, USA) with FEI-accelerated voltage of 20.00 kV. The observations were performed on the studied samples without any preparation at low vacuum. The ESEM analysis was conducted in the Grand Egyptian Museum—Conservation Center (GEM.CC), Cairo, Egypt.

##### Measurement of Cellulose Crystallinity by XRD

X-ray diffraction analysis was used for the determination of cellulose crystallinity of new and historical paper samples using Panalytical X, Pert pro-PW 3040/60—Scan axis: Gonio—Anode material: Cu—Generator settings: 40 mA, 45 kV—Goniometer radius: 240 mm at the Grand Egyptian Museum—Conservation Center (GEM.CC), Egypt. The crystallinity of cellulose was determined from the crystallinity index (%), which was calculated according to the following Equation [11]:

(1) $$I_{\mathrm{crys}}=\frac{I_{002}-I_{\mathrm{am}}}{I_{002}}\times 100$$

where I_Crys_ is the crystallization index, I_002_ is the intensity at a 2θ value of 22.6°, and I_am_ is the intensity at a 2θ value of 19°.

#### 2.2.2. Fungal Isolation and Identification

Sterilized cotton swabs were used to collect samples from the damaged area, which were then placed directly into a sterile tube and sent to the laboratory. These samples were used as a source for isolating various fungal strains associated with the historical manuscript. To increase or reactivate the fungal spore, the collected swabs were introduced separately into the test tube containing 10 mL of sterilized saline solution supplemented with an antibacterial agent (chloramphenicol, 500 mg L^−1^) for 24 h at 252 °C. The Czapeck Yeast Extract (CYA) agar media (ready prepared, Merck, Rahway, NJ, USA) and potato dextrose agar media (ready prepared, Oxoid, Hampshire, UK) were then covered with 100 L of the preceding saline solution and incubated at 25 °C. The inoculated plates were observed daily, and the growing fungal colony was picked up and reinoculated over new agar plates for future purification.

The acquired fungal isolates were subjected to primary identification using microscopic and cultural traits based on standard keys for *Cladosporium* spp. [12], *Aspergillus* spp. [13], and *Penicillium* spp. [14].

After that, the isolated fungal strains were molecularly identified using the ITS sequence analysis. To confirm their identity, six fungal strains (one from each strain) were chosen based on traditional identification. The technique for the Gene Jet Plant genomic DNA purification Kit (Thermo, Waltham, MA, USA) was used to extract fungal DNA.

Fungal DNA served as a template for the PCR that amplified the ITS region. The ITS1 and ITS4 primers (5 TCCGTAGGTGAACCTGCGG-3 and 5 TCCTCCGCTTATTGATATGC-3, respectively) were used. Maxima Hot Start PCR Master Mix (Thermo), 0.5 M of each primer, and 1 L of isolated fungal DNA were added to 50 L of the PCR mixture. According to Fouda et al. [15], the PCR method’s protocol was successfully executed. Using the NCBI-BLAST tools, the resulting sequences were compared to ITS sequences deposited in the GenBank. The phylogenetic tree was built using the neighbor-joining method with confidence-tested bootstrap analysis over 1000 iterations (MEGA v6.1, www.megasoftware.net, 19 January 2013).

#### 2.2.3. Enzyme Activity

Using the agar plate method, the efficacy of different fungal strains isolated from deteriorated historical manuscripts to secrete different enzymes including cellulase, amylase, gelatinase, and pectinase was evaluated. A fresh disc (0.5 mm in diameter) of each purified fungal strain was added in the center of a mineral salt agar (MSA, containing: KCl, 5 g L^−1^; NaNO_3_, 6 g L^−1^; MgSO_4_.7H_2_O, 0.5 g L^−1^; KH_2_PO_4_, 1.5 g L^−1^; ZnSO_4_, 0.01 g L^−1^; FeSO_4_, 0.01 g L^−1^; agar, 15 g L^−1^, Dis. H_2_O, 1.0 L) plate. The MSA media were supplemented with 1% of a particular substrate for each enzyme, which were carboxymethyl cellulose for cellulase enzyme, starch for amylase, gelatin for gelatinase, and pectin for pectinase. To prevent bacterial growth, chloramphenicol, an antibacterial agent, was added to the MSA media. After inoculation, the plates were incubated at 25 °C for 96 h. At the end of the incubation period, the plates containing carboxymethyl cellulose, starch, and pectin were flooded with an iodine solution to detect the cellulase, amylase, and pectinase activity, respectively. On the other hand, the plate containing gelatin was flooded with acidic mercuric chloride for the detection of gelatinase activity [16].

The results (mm) were recorded as follows [17]:

Diameter of the entire clear zone (mm) − diameter of the growth of the fungal colony (mm)

#### 2.2.4. Biocontrol of Fungal Growth by Probiotic Strain

##### Probiotic Bacterial Strain

We evaluated the potential of *Lactobacillus rhamnosus* ATCC-7469 (a probiotic strain) metabolites to biocontrol fungal strains. This strain was purchased from the Microbiological Resources Centre (MIRCEN), Faculty of Agriculture, Ain Shams University, Cairo, Egypt.

##### Bacterial Growth and Extraction

The *L. rhamnosus* strain was inoculated in 100 mL of MRS broth media and incubated under anaerobic conditions at 35 ± 2 °C for 72 h. After that, the inoculated broth media were centrifuged at 10.000 rpm for 10 min to collect the supernatant, which was subjected to filtration using a filter with pore sizes of 0.22 µm to prepare the cell-free supernatant (CFS) [18]. For extraction, 100 mL of collected CFS was mixed with 100 mL of ethyl acetate for 20 min in a separation funnel, which stood at room temperature until two distinct layers formed. The extraction process was conducted twice to ensure that all metabolites were extracted. During each extraction step, the organic phase was gathered and then concentrated using a rotary evaporator. The resulting pellets were then collected, dried, and dissolved in DMSO for further investigation of their biocompatibility and antifungal activity [19].

##### Gas Chromatography–Mass Spectrometry (GC-MS) Analysis Conditions

The qualitative and quantitative bioactive secondary metabolites in the collected organic phase were evaluated using GC-MS (Agilent Technologies, Santa Clara, CA, USA). In the GC-MS system, a mass spectrometer detector (5977A) and gas chromatography (7890B) were used. The GC system was equipped with a column of HP-5MS with an internal diameter of 30 m × 0.25 mm and a film thickness of 0.25 m. Moreover, the hydrogen was employed as a carrier gas during the analysis, with a 1 mL/min flow rate at a splitless injection volume of 1 µL. The temperature program was set up as follows: 50 °C for 1 min; 5 °C/min rise to 100 °C and hold for 0 min; 10 °C/min rise to 300 °C and hold for 5 min. Additionally, the temperature of the detector and injector was adjusted to 260 °C and 250 °C, respectively. With a 6 min solvent delay, the mass spectra in the range of 50–550 *m*/*z* were produced at an electron ionization of 70 eV. The constituents of the sample were identified by comparing the obtained fragmentation pattern with those deposited in the NIST Mass Spectral Library and Wiley [20].

##### Biocompatibility of Extracted Metabolites

The biocompatibility of ethyl acetate extract and two normal cell lines designated as human fibroblast lung tissue (WI38) and human normal melanocytes (HFB4) was investigated using the MTT (3-(4,5-dimethylthiazol-2-yl)-2,5-diphenyl tetrazolium bromide) assay method. The selected cells were purchased from the Holding Company for Biological Products and Vaccines (VACSERA), Cairo, Egypt.

Each cell was inoculated in a 96-well culture plate with the intensity of 1 × 10^5^ cells/100 µL/well and incubated at 37 °C for 24 h in a CO_2_ incubator (5%). At the end of the incubation time, the formed monolayer sheet was mixed with 100 µL of RPIM media in presence of 2% serum. After that, the growing cells were treated with different concentrations (1000–31.25 μg mL^−1^) of ethyl acetate extract after evaporating the solvent and dissolving the residue in DMSO. The treated plates were incubated at 37 °C for 48 h. In each plate, three wells without treatment were used in the experiment as a control. After 48 h, the excess growth media in each well were removed and filled with 50 µL of MTT solution (5 mg mL^−1^ phosphate buffer saline solution), shaken well for 5 min, and incubated at 37 °C for 4 h. After that, the excess MTT solution in each well was removed, followed by the wells being filled with 100 µL of DMSO (10%) for dissolving the formazan crystal that formed because of the MTT assay metabolism. The DMSO was removed from the wells after 30 min and the absorbance of the formed color was measured using an ELIZA reader, producing 570 nm [21]. The cell viability percentages (%) due to treatment with crude probiotic extract were detected using the equation as follows:

(2) $$\mathrm{Cell}\;\mathrm{viability}\;\mathrm{percentages}\;(\%)=\frac{\mathrm{Absorbance}\;\mathrm{of}\;\mathrm{treatment}}{\mathrm{Absorbance}\;\mathrm{of}\;\mathrm{control}}\times 100$$

##### Antifungal Activity of the Probiotic Crude Extract

To investigate the antifungal activity of the crude ethyl acetate extract in inhibiting the growth of the isolated fungal strains, the well diffusion method was employed. The concentrations used for the antifungal activity assay were lower than the IC_50_ value, which was determined by biocompatibility assay. Various concentrations of the crude extract, including 300, 200, 100, 50, and 25 μg mL^−1^, were used. For this method, 50 µL of spore suspension from each fungal strain (adjusted the OD at 1) was spread thoroughly using a sterilized swab over the surface of Czapeck Yeast Extract Agar media. After that, four wells (0.6 mm in diameter) in each plate were taken and filled with 100 µL of each concentration. The loaded plates were kept in the refrigerator for 1 h before being incubated at 27 ± 2 °C for 48 h. The solvent system (DMSO) was used as a control. The results were recorded as a diameter of the clear zone (mm) formed around each well [22]. The lowest concentration of crude extract that forms the clear zone was detected as the minimum inhibitory concentration (MIC) [23]. The experiment was performed in triplicate.

#### 2.2.5. Statistical Analysis

The means of three independent replicates were used to represent the data in the current study, which were then subjected to an ANOVA analysis with the statistical package SPSS v17. The Turkey HSD test, with a *p* value of 0.05, was used to examine the mean difference comparison between the treatments.

## 3. Results and Discussion

### 3.1. Analytical Techniques Used for Condition Assessment of the Studied Manuscript

#### 3.1.1. Visual Assessment

**Figure 1:**
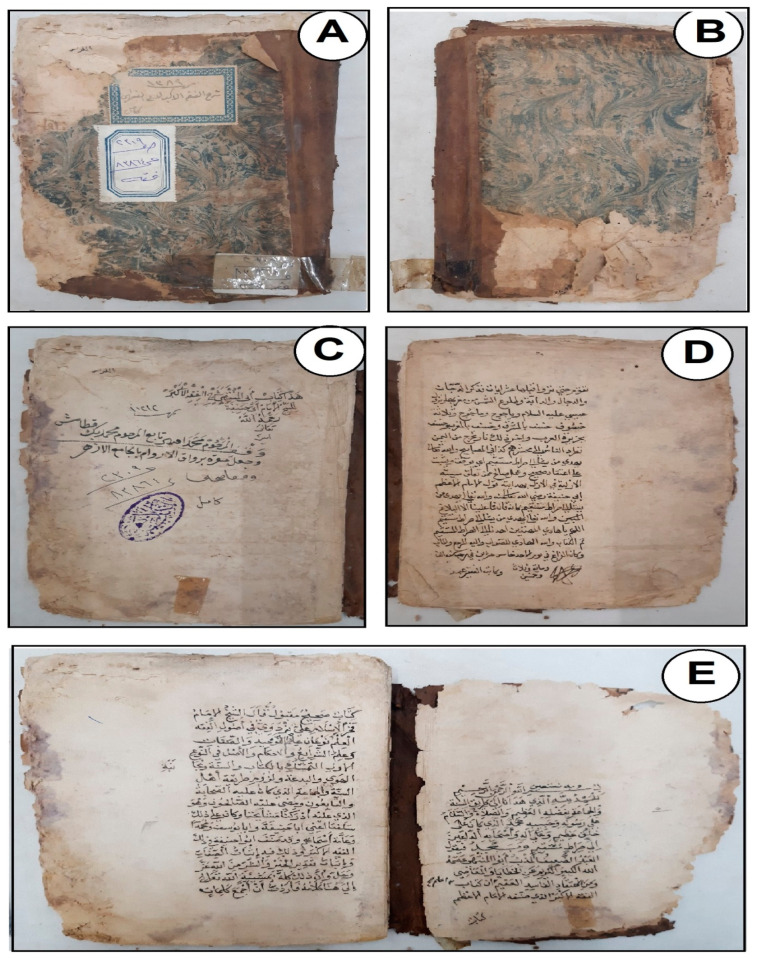
Some deterioration aspects of the components of the historical manuscript studied at Al-Azhar Library, Cairo, Egypt: (**A**,**B**) Leather binding, (**C**–**E**) Paper sheets.

Visual assessment is becoming vital in the conservation field [24]. It was demonstrated that the paper and leather binding of our manuscript had undergone significant deterioration, and some of the manifestations were visible on the surface of the manuscript’s components (Figure 1). These aspects include hardness, loss of flexibility, erosion of the tanning substance, loss of various portions, the presence of dust, and some stains derived from different sources, such as pollution or fungal contamination. Furthermore, one of the key signs of leather binding deterioration is the existence of certain labels attached to the pressure-sensitive tape. Furthermore, shrinkage and color variability (some areas are dark, while others are less dark) were noted (Figure 1A,B). It can be concluded that these deterioration manifestations may have arisen due to unfavorable external environmental conditions, which are responsible for most of the degradation factors mentioned earlier. These conditions have also facilitated the growth of fungal strains. Numerous published works have reported that unregulated environmental conditions, including light, humidity, temperature, and pollutants, are primarily responsible for the majority of the deterioration observed in historical manuscripts [25,26]. According to Sebestyén et al. [27], there are both internal and external causes of leather degeneration. The manufacture of leather artifacts is what causes the interior variables, while the external factors are brought on by biological factors (such as microorganisms and insects) and natural events, such as those brought on by climate change, which are due to environmental factors (such as light irradiation, relative humidity, temperature, and chemical and particulate matter pollution). According to research by Carsote et al., changes in temperature and relative humidity (RH) cause the fibers of paper and vegetable-tanned leather to shrink [28]. The preservation conditions of the studied manuscript, as mentioned in the “Materials and Methods” section, particularly the RH (60%) and temperature (27 °C), are favorable for the growth of different microbes such as fungi, actinomycetes, and bacteria, which are key drivers of biodeterioration [29].

Certain types of deterioration aspects were seen in the historical paper manuscript (Figure 1C–E), including yellowing of the paper, weakness, fragility, tears, deformation of the surface, and brittleness. One of the first indications of paper’s disintegration and age is its yellowing. The yellow and brown colors appeared in different parts of paper and leather binding, which may be due to the microbial contaminations that cause the manuscript components to become brittle and lose their mechanical properties and esthetic value [30,31]. Furthermore, when cellulose is subjected to unfavorable environmental conditions, it can produce acids such as lactic, formic, oxalic, and acetic acids, leading to more deterioration [26]. As a result of the strong intermolecular bonds, the acids present in paper are not readily released, but the moisture content causes the glucose chains to progressively shorten. The hydrolysis reaction that occurs leads to the generation of additional acids, which further accelerates paper’s degradation process.

It is worth noting that microbial contamination can also be a contributing factor to the discoloration of paper, as previously mentioned. In our latest study, reddish-brownish foxing patches were caused by fungi and bacteria identified on historical manuscripts from the 17th century [15]. Additionally, the purple spots were caused due to contamination with colored mold strains such as *Epicoccum* sp. and *Monoascus* sp., which thrive in environments with inadequate aeration, RH, and temperature [32].

#### 3.1.2. Investigation of the Surface Morphology by Scanning Electron Microscope (SEM)

**Figure 2:**
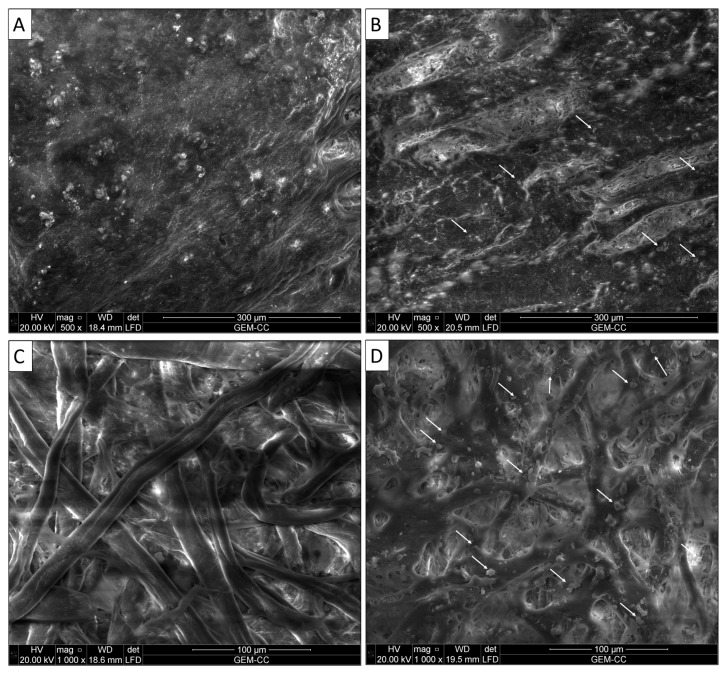
SEM images of paper and leather binding of the studied manuscript: (**A**) New vegetable-tanned leather binding, (**B**) historical leather binding, (**C**) Whatman paper (control), (**D**) historical paper. The arrows refer to microbial growth.

The data obtained showed that the grain layer of the new vegetable-tanned leather sample was smooth, and fine follicles were also clearly visible (Figure 2A). There was also a clear grain surface pattern on the skin characteristic of goatskin. The surface of the historical leather binding was coarse, the grain surface could not be recognized, and the type of animal used in the manufacture of the leather binding was difficult to identify (Figure 2B). All aspects of deterioration found on the surface were due to improper handling and environmental conditions [33]. The results also showed that there were some fine bores, which can be attributed to insect invasion [34].

For the Whatman paper, the results showed that the fibers are ribbon-shaped, with some twists in the shape of spirals that alternately point to the right and left and appear to be strong and broad (Figure 2C), which suggested that the fibers of the paper manuscript were made of cotton. The fiber structure of the new sample indicated the strength and durability of the fibers and their good distribution. The results of the surface morphology of the historical paper showed that the fibers were made of cotton. The weakness and random distribution of the fibers were noticed. This may be due to the improper environmental conditions mentioned above (Figure 2D). Interestingly, the mycelium, hyphae, and spores of fungi were seen among the cotton fibers as short rods (bacteria) or globes (fungal growth) [35]. Environmental surrounding conditions, especially RH, temperature, and pollution, are the main reasons for the susceptibility of the paper to the growth of various fungal strains [25].

#### 3.1.3. X-ray Diffraction Analysis (XRD) for Measurement of Cellulose Crystallinity

**Figure 3:**
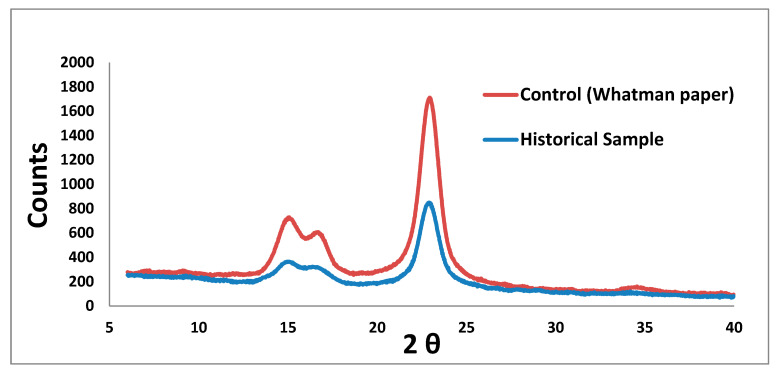
X-ray diffraction analysis for measurement of new and historical paper crystallinity.

Native cellulose is recognized to possess a crystalline structure. Determining cellulose crystallinity is regarded as one of the most significant properties of cellulosic materials, as it provides insight into their mechanical, physical, and chemical capabilities. Within the amorphous and crystalline sections of the fiber cell wall, the cellulose molecules are organized in fibrils [36]. The data obtained showed that the crystallinity index of the Whatman paper sample (control) was 82%, and for the historical paper it was 69% (Figure 3). It was evident that changes in the environment, particularly an increase in relative humidity, had an impact on the crystallinity of cellulose. Because the Whatman paper contains more than 95% cellulose, it has a high degree of crystallinity in general [37]. The present investigation revealed that the crystallinity of the historical paper was significantly lower in comparison to the control sample. This reduction can be attributed to various deterioration factors, including the rising temperature and decreasing moisture content, which lead to the damage and destruction of the amorphous regions of cellulose. Additionally, microbial activity can contribute to the formation of fossilized and hardened papers [38,39].

### 3.2. Fungal Isolation and Identification

Fungi are considered the main source for the biodeterioration of historical manuscripts because the subject offers a source of nutrients and carbon that supports the growth of fungi [40]. Among fungal deterioration, symptoms are increasing brown spots, brittleness, discoloration, and weakening of the paper [25]. In the current study, culture-dependent methods were utilized to isolate fungi from the swabs collected after being pressed onto highly deteriorated areas. Twelve fungal strains were obtained from the collected swabs and subjected to identification at first based on morphological, cultural, and microscopic examination. According to standard keys, these fungal strains were identified as *Cladosporium* spp. (two isolates coded as AF-1 and AF-2), *Aspergillus fumigatus* (five isolates coded as AF-3–AF-6, and AF-8), *A. ustus* (one isolate coded as AF-7), *A. flavus* (two isolates coded as AF-9 and AF-10), *A. niger* (one isolate coded as AF-11), and *Penicillium chrysogenum* (one isolate coded as AF-12). In a similar study, 31 fungal strains were isolated from an old manuscript collected from the Universitas Indonesia Library and classified based on morphological identification into *Aspergillus* spp. (fourteen isolates), *Penicillium* spp. (five isolates), *Cladosporium* spp. (eight isolates), *Curvularia* sp. (one isolate), *Ulocladium* sp. (one isolate), and yeast-like fungi (two isolates) [40]. Additionally, the most common fungal strains associated with deteriorated historical manuscripts dated back to the 17th century belong to *Aspergillus flavus* and *Aspergillus niger* [41]. Moreover, the *Aspergillus* and *penicillium* species were the most common fungal strains isolated from 79 deteriorated old manuscripts collected from Astan-Quds Library, Mashhad, Iran [42]. Oetari et al. [43] reported that the fungal strains *A. flavus*, *A. niger*, *A. versicolor*, *A. fumigatus*, *A. ruber*, *A. clavatus*, and *P. citrinum* were isolated from a deteriorated manuscript collected from Indonesia. *Cladosporium* spp. such as *C. colocasiae* and *C. herbarum* were previously isolated from deteriorated historical Chinese manuscripts collected from the Central Library, Indonesia, and those collected from Mertasinga, Cirebon [44,45].

As shown, the *Aspergillus* spp. represented 75% of the total fungal strains isolated in the current study, followed by *Cladosporium* spp. with 16.7%, and *Penicillium* sp. with 8.3%. Among *Aspergillus* spp., *A. fumigatus* represented the highest percentage of 55.6%, followed by *A. flavus*, *A. ustus*, and *A. niger* with percentages of 22.2, 11.1, and 11.1%, respectively. Incompatible with the obtained results, *Aspergillus* spp., followed by *Penicillium* spp., *Eurotium* spp., and *Sterile hyphae*, are the most common fungal strains isolated from European historical manuscripts dated back to the 19th century, with percentages of 45, 35, 5, and 15%, respectively [9]. Recently, *Aspergillus* spp., *Penicillium* spp., *Induratia* spp., and *Paecilomyces* spp. were the most common fungal strains isolated from historical papers dating back to the 14th century, with percentages of 42.8, 42.8, 7.1, and 7.1%, respectively [26]. The authors reported that *A. ustus* followed by *A. terreus* and *A. chinensis* represented the most common *Aspergillus* species, with percentages of 50, 33.3, and 16.6%, respectively. Meanwhile, *P. citrinum* and *P. chrysogenum* represented the most common *Penicillium* species, with percentages of 66.6 and 33.4%, respectively (these percentages were calculated based on the total number of *Aspergillus* spp. and *Penicillium* spp.).

Fungi can penetrate the paper fibers, forming a hyphal network that leads to destroying the materials via hydrolytic enzyme degradation, acid corrosion, and mechanical attack [46]. Pigmented secondary metabolites secreted by fungi that inhabit fibers can cause the yellowing and discoloration of paper [47]. The secretion of melanin pigment by various fungal strains may be one of the main causes of brown spots on deteriorated papers [8]. The growth of *Penicillium chrysogenum*, *P. commune*, *P. citrinum*, *Chaetomium murorum*, *C. globosum*, *Stachybotrys chartarum*, *Myxotrichum deflexum*, and *Eurotium rubrum* is responsible for the presence of deep brown, black, brown, and light orange spots on the deteriorated papers [48]. The majority of fungal strains associated with deteriorated papers are mostly airborne. Fungal growth on old papers can promote the growth of bookworms such as *Liposcelis bostrychophila*, which feeds on the organic compounds resulting from the degradation caused by fungi [40,49]. The presence of this bookworm in old manuscripts can cause irregular holes in deteriorated samples or convert it into powder [50].

**Figure 4:**
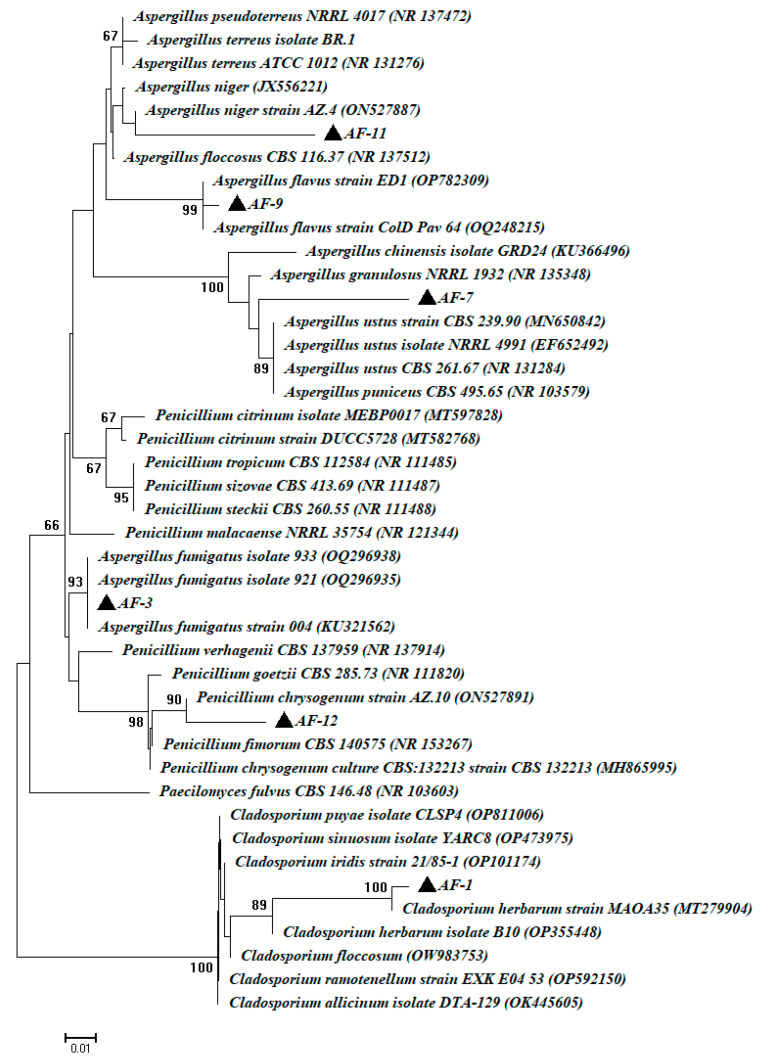
Phylogenetic tree of selected fungal strains isolated from a deteriorated manuscript dated back to the 16th century. This tree was constructed using the neighbor-joining method, bootstrap value of 1000 replicates.

To confirm the traditional identification, one strain from each species was selected for ITS sequence analysis. For this goal, fungal strains designated AF-1, AF-3, AF-7, AF-9, AF-11, and AF-12 were selected (representing all fungal species found) for ITS identification. The sequence analysis showed that the fungal strain AF-1 was similar, with a percentage of 99.98%, to *Cladosporium herbarum* (closest accession number: OP355448). However, the fungal strains AF-3, AF-7, AF-9, and AF-11 were similar to *Aspergillus fumigatus*, *A. ustus*, *A. flavus*, and *A. niger* (closest accession numbers were OQ296935, MN650842, OM283738, and JX556221, respectively) with percentages of 98.98, 99.42, 99.89, and 98.28%, respectively. On the other hand, the fungal strain AF-12 was matched with *Penicillium chrysogenum* (closest accession number: ON527891), with a similarity percent of 98.89%. Therefore, the selected fungal strains were identified as *C herbarum* strain AF-1, *A. fumigatus* strain AF-3, *A. ustus* strain AF-7, *A. flavus* strain AF-9, *A. niger* strain AF-11, and *P. chrysogenum* strain AF-12 (Figure 4). The six sequences obtained in the current study were deposited in GenBank under the accession number OQ564401–OQ564406.

The isolation and identification of different fungal strains related to old papers in museums, libraries, and archives serves not only to explore their role in biodeterioration but also to protect the health of visitors and workers against negative impacts caused by these fungi. For instance, the *Aspergillus* species produces a mycotoxin substance that has hepatocarcinogenic properties [51]. Additionally, *Cladosporium herbarum* and *C. cladosporioides* produce specific proteins that have allergic properties [52]. Moreover, *A. niger*, *A. terreus*, *A. flavus*, *A. ustus*, *A. udagawae*, *A. quadrilineatus*, *A. alliaceus*, *P. chrysogenum*, and *P. citrinum* have the capacity to produce different metabolites that cause aspergillosis, otomycosis, endocarditis, cutaneous inflammation, dermal infection, phaeohyphomycosis, allergies, and respiratory infections [40,53,54].

### 3.3. Enzymatic Activities

**Figure 5:**
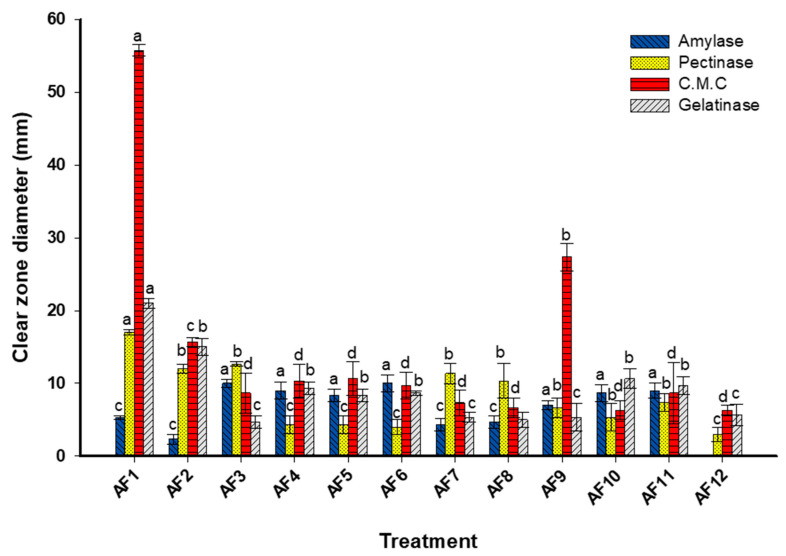
Hydrolytic enzyme activity of cellulase, amylase, pectinase, and gelatinase (mm) of different fungal strains isolated from the deteriorated historical manuscript. Different letters on bars for the same enzyme indicate that the mean values of the clear zone are significantly different (*p* ≤ 0.05) (*n* = 3).

In the current study, the fungal strains identified from the deteriorated samples were found to have the capability to secrete a range of hydrolytic enzymes that are responsible for breaking down the primary components of paper and leather. As shown, all fungal strains have the capacity to secrete various detected enzymes (amylase, cellulase, pectinase, and gelatinase) except fungal strain *P. chrysogenum* AF-12, which lacks amylase activity (Figure 5). The highest cellulase activity was recorded for fungal strains *C herbarum* AF-1 and *A. flavus* AF-9, with clear zones of 55.8 ± 1.5 mm and 27.3 ± 3.2 mm, respectively (Figure 5). However, the lowest cellulase production was recorded for the fungal strains AF-12, AF-10, and AF-8, with clear zones of 6.1 ± 1.2, 6.3 ± 2.3, and 6.7 ± 2.2 mm, respectively. On the other hand, the maximum amylase productivity was recorded for fungal strain AF-3, with a clear zone of 10.1 ± 1.0 mm, followed by fungal strain AF-4, with a clear zone of 9.0 ± 2.2 mm. The fungal strain AF-1 was recorded as the highest producer of pectinase and gelatinase, with a clear zone of 17.0 ± 0.8 and 21.0 ± 1.4 mm, respectively. Overall, the cellulase enzymes showed the highest enzymatic activity, suggesting that the identified fungal strains are involved in the biodegradation of cellulose, the main component of paper.

Sterflinger and Pinzari reported that the most common fungal strains isolated from deteriorated old papers that have the efficacy to secrete a wide variety of degrading enzymes were the *Cladosporium*, *penicillium*, and *Aspergillus* species [55]. In addition to the main components of papers, some additives such as proteins, sugars, gelatin, and starch flours were added to decrease the spread of ink and improve the attachment of fibers to each other [6]. The secretion of various hydrolytic enzymes leads to the degradation of paper components in addition to additives, and ultimately to the weakening and deterioration of historical manuscripts. Data recorded by El-Bergadi et al. were incompatible with our obtained data; they mentioned that among the 31 fungal strains isolated from historical papers deposited in Medina of Fez, only 9 strains had the ability to produce cellulase enzymes [56]. Additionally, among the 28 fungal strains obtained from the National Archive Republic of Cuba, 88%, 54%, and 81% of the total fungal strains had the potential to secrete cellulase, gelatinase, and amylase enzymes [57]. Savković et al. [58] reported that the *Penicillum* and *Aspergillus* species were the most common strains obtained from culture heritage deposited in Serbia. The authors mentioned that 47% (16 isolates) and 82% (28 isolates) of the total of 34 fungal strains had the ability to produce protease and cellulase enzymes. Cellulose and starch, which are large molecules, were broken down into smaller units to form glucose monomers through the action of cellulase and amylase enzymes. Moreover, different protein sources such as fibroin, collagen, and keratin incorporated into parchment, wool, leather, and silk were degraded by the action of gelatinase (protease) enzyme-forming small subunits [6].

### 3.4. Biocontrol of Fungal Strains by Cell-Free Filtrate (CFF) of Probiotic Bacterial Strain L. rhamnosus

#### 3.4.1. Metabolic Profile Analysis Using Gas Chromatography–Mass Spectrometry (GC-MS)

**Figure 6:**
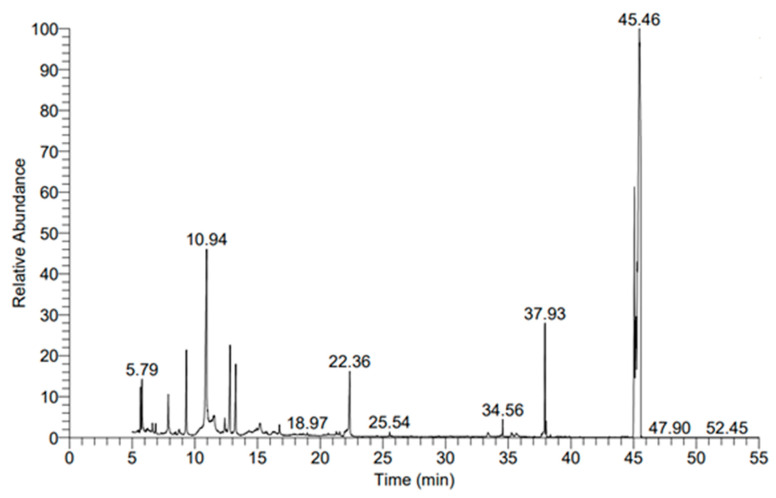
GC-MS chart for CFF of probiotic bacterial strain *L. rhamnosus*.

The antifungal activity of probiotic bacterial strains is associated with their metabolites secreted in the CFF (surrounding media) [59]. Therefore, the metabolic profile of the CFF of probiotic bacterial strain *L. rhamnosus* was detected by GC-MC analysis (Figure 6). As shown, the metabolic profile of *L. rhamnosus* contains > 50 metabolites based on the Wiely-9 and nist_ms libraries. These various metabolites were detected at RTs of 5. 67, 5.79, 6.87, 7.88, 9.32, 10.93, 11.57, 12.39, 12.81, 13.26, 15.2, 16.75, 22.35, 34.56, 37.93, 45.06, 45.14, 45.2, 45.29, and 45.49 (Figure 6, Table 1). Among these metabolites, different chemical compounds have low and high molecular weights, such as amides, acids, ethers, esters, alcohol, and carboxylic acids (Table 1). For instance, various acids such as lactic acid, propanoic acid, acetic acid, phenyllacetic acid, and hexa- and octadecanoic acid were observed. Moreover, esters such as methyl and ethyl, and butyl ester, were present in CFF. Various alcohols and amides were also present (Table 1). According to the published literature, different compounds that exist in CFF have various activities such as antifungal, anti-inflammatory, cytotoxicity, and food safety. For instance, phenyllacetic acid at an RT of 16.75 was reported as a promising antifungal agent for *Aspergillus* and *Penicillium* spp. [60]. Shehata et al. [59] reported that the metabolites secreted by *Lactobacillus* are strain dependent, which means the metabolites, and hence their activity, differ between various *Lactobacillus* strains.

#### 3.4.2. Biocompatibility of Metabolites of Probiotic Bacterial Strain

**Figure 7:**
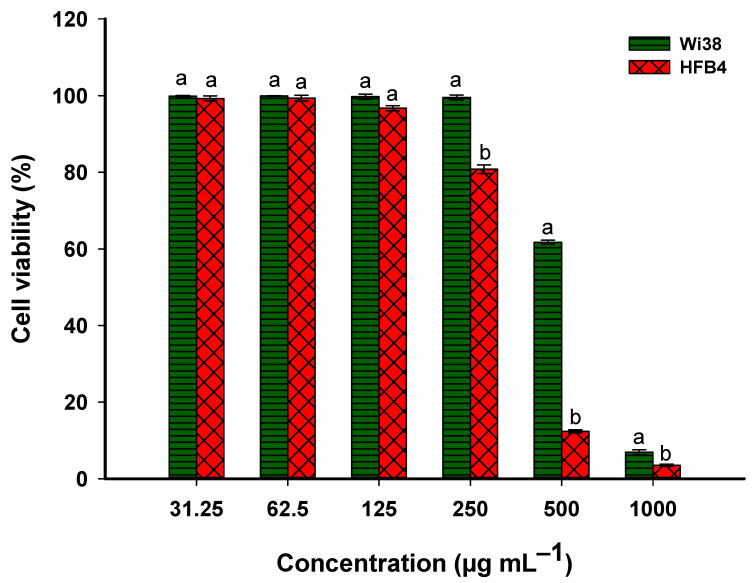
Biocompatibility of cell-free filtrate of *L. rhamnosus* with two normal cell lines, Wi23 (normal lung tissue) and HFB4 (normal human skin cells). Different letters on bars for the same concentration indicate that the mean values are significantly different (*p* ≤ 0.05) (*n* = 3).

The MTT assay was used to assess the biocompatibility of metabolites secreted by the probiotic *L. rhamnosus* strain with two normal cell lines, Wi38 and HFB4. Various dilutions of the bacterial metabolites were prepared and evaluated for their effectiveness on the normal cells. As shown, the metabolites of the *L. rhamnosus* strain had low effects on the proliferation of the two normal cells. The viability of the Wi38 cells was decreased from 99.9 ± 0.3% to 61.7 ± 0.9% in a dose-dependent manner by metabolites from 31.25 to 500 µg mL^−1^. Meanwhile, the cell viability of HFB4 was decreased from 99.3 ± 1.2% to 80.8 ± 1.9% at a concentration in the range of 31.25 to 250 µg mL^−1^ (Figure 7). In a similar study, metabolites of *L. lactis* had a lower cytotoxic effect on the viability of the HUVEC normal cell line, with percentages of 99 to 78% at concentrations in the range of 0.39–25 µg mL^−1^ [61]. Additionally, the metabolites secreted by probiotic bacterial strains *L. acidophilus* and *Bifidobacterium* sp. did not exhibit a cytotoxic effect on normal fibroblast GM07492-A cell lines [62]. The normal cell line MCF-10A was found to be unaffected by the different dilutions of the cell-free filtrate of *L. acidophilus* GG and *L. acidophilus* LA-5, in contrast to the high cytotoxic effects observed in the same dilutions on the cancer cell lines MCF-7 and Caco-2 [63]. Moreover, the authors reported that the low dilution of probiotic cell-free filtrate enhances the viability of normal cells.

The detection of IC_50_ (the dilution that inhibits the viability of 50% of normal cells) is a critical parameter that should be detected before use in biocontrol. Herein, the IC_50_ value of cell-free metabolites against two normal cells, Wi38 and HFB4, were 525.2 ± 9.8 and 329.1 ± 4.2 µg mL^−1^. According to the published literature, the metabolites of probiotic bacterial strains targeted cancer cells at low concentrations compared to normal cells. For instance, the survival of the breast cancer cell, MCF-7, after being treated with nisin (secreted by *L. lactis*) was highly inhibited at low concentrations, with IC_50_ of 17 µg mL^−1^, compared with the IC_50_ of a normal cell (HUVEC), which was 64 µg mL^−1^ [61]. The high activity of probiotic metabolites against cancer cells compared to normal cells could be related to the differences in cell membrane structure. Cancer cells are typically characterized by the presence of anionic molecules, such as phosphatidylserine, on their cell membranes, which gives their cell surfaces a negative charge [64]. However, the surface of normal cells usually has a neutral charge due to the presence of zwitterionic lipids such as phosphatidylethanolamine, sphingomyelin, and phosphatidylcholine [65]. The majority of probiotic bacterial metabolites are characterized by their hydrophobic and cationic structures [66]. The reason for the high cytotoxic effect of the cell-free filtrate may be the electrostatic attraction between the anionic cancer cell membrane and the cationic metabolites. Additionally, according to some of the published literature, the cytotoxic potential of probiotic metabolites may be due to their ability to alter the concentrations of intracellular membrane ions, create pores and disrupt the cell membrane structure, induce cell cycle arrest, alter transmembrane gradients, and trigger apoptosis. These effects are more prominent in cancer cells than in normal cells [61,62,67]. In the current study, we used concentrations below IC50 to investigate their antifungal activities against different fungal strains obtained from deteriorated historical manuscripts to recommend them for use in the biotreatment and preservation of old papers against fungal deteriorations.

#### 3.4.3. Antifungal Activity

Probiotic bacteria have the ability to produce a wide variety of active compounds such as organic acids, cyclic peptides, phenolic compounds, H_2_O_2_, and diacetyl [68]. The usage of these compounds is dependent on their chemical characteristics. For instance, the peptide compounds secreted by probiotic bacteria are preferred due to their high thermal and pH stability [69]. Recently, metabolites produced by *Lactobacillus* sp. were used to inhibit the growth of phytopathogens that infect maize seeds and wheat grain during their storage period [70]. The evaluation of the potential of metabolites produced by *L. rhamnosus* to inhibit fungal growth responsible for the deterioration of historical papers, as a potential preservation method, was applied during the current study. For this purpose, different concentrations (300, 200, 100, and 50 µg mL^−1^) of *L. rhamnosus* metabolites were used.

**Figure 8:**
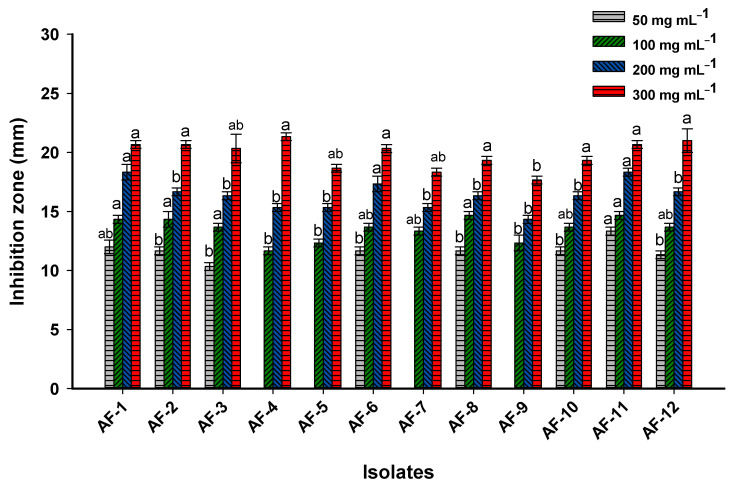
Antifungal activity of CFF of *L. rhamnosus* against twelve fungal strains isolated from the historical manuscript. Different letters on bars at the different concentrations indicate that the mean values of the inhibition zone are significantly different (*p* ≤ 0.05) (*n* = 3).

Data analysis showed that the antifungal activity of cell-free filtrate occurred in a concentration-dependent manner. As shown, the highest activity against all isolated fungal strains was recorded at a concentration of 300 µg mL^−1^. At this concentration, the maximum activity was recorded toward fungal strain *A. fumigatus* AF-4 with a zone of inhibition (ZOI) of 21.3 ± 0.6 mm, followed by the fungal strains *C. herbarum* AF-1, *C. herbarum* AF-2, and *A. niger* AF-11, with a ZOI of 20.7 ± 0.6 mm (Figure 8). The lowest activity of CFF at this high concentration was shown toward fungal strain *A. flavus* AF-9, followed by fungal strain *A. ustus* AF-7 and A. fumigatus AF-5, with ZOIs in the range of 17.7 ± 0.5 to 18.7 ± 0.6 mm. The antifungal activity was decreased at low CFF concentrations. For instance, although the activity of 100 µg mL^−1^ of CFF was still against all fungal strains, the ZOIs were decreased to the range of 11.7 ± 0.6 mm (for fungal strain *A. fumigatus* AF-4) to 14.7 ± 0.6 mm (for fungal strains of *A. fumigatus* AF-8 and *A. niger* AF-11) (Figure 8). Moreover, the CFF at a concentration of 50 µg mL^−1^ was active against 66% of the total fungal strains. The activity at this low concentration was lost toward fungal strains of *A. fumigatus* AF-4, *A. fumigatus* AF-5, *A. ustus* AF-7, and *A. flavus* AF-9 (Figure 8). The MIC value for CFF was 25 µg mL^−1^ for fungal strains AF-1, AF-2, AF-3, AF-6, AF-8, AF-10, AF-11, and AF-12. However, the concentration of 100 µg mL^−1^ of CFF represented the MIC value for fungal strains AF-4, AF-5, AF-7, and AF-9.

The antifungal mechanism of metabolites of *Lactobacillus* spp. is thought to involve several mechanisms. One mechanism is the production of organic acids, such as lactic acid and acetic acid, which can lower the pH of the environment and inhibit the growth of fungi. The antifungal activity of CFF of *L. sanfrancisco* could be the presence of various acids such as acetic acid, Caproic acid, n-valeric acid, Butyric acid, propionic acid, and formic acid [71]. Additionally, the CFF of *L. plantarum* showed antifungal activity toward *A. parasiticus* with a ZOI of more than 12 mm due to the presence of acids (oleic, lactic, and octanoic acids), esters (long-chain), p-dioxane-2,3-diol, and butanamide [72]. In the current study, the CFF of *L. rhamnosus* contained various acids such as acetic, lactic, oxalacetic, propanoic, (hexa- and octa-) decanoic, and benzoic acid (Table 1). These acids create an acidic environment that leads to the restriction of cell growth or cell deformation [71]. studies, phenyllacetic acid, which is one of the components of CFF in the current study (RT = 16.75), was reported to have promising antifungal activity against various strains of *Aspergillus* and *Penicillium* [60].

Another mechanism involves the synthesis of bacteriocins, which are tiny peptides with antimicrobial properties capable of either killing or inhibiting the growth of fungi. The high antifungal activity of the CFF of *L. amylovorus* strain DSM 19,280 against *A. fumigatus* can be attributed to the presence of seventeen different compounds, including two nucleosides, five cyclic peptides, nine carboxylic acids, and sodium decanoate [73]. Some symptoms that appear in *Fusarium proliferatum* after treatment with CFF of *L. plantarum* were disrupted, wrinkled, deformed, twined, and shrunken, and it resulted in the appearance of conglobated tips [74]. Rao et al. [72] reported that the hyphal cell wall of *A. parasiticus* was damaged due to treatment with CFF of the *L. plantarum* strain MYS44, causing a discharge of cytoplasmic contents to the outside and, hence, cellular destruction.

In addition, some metabolites produced by *Lactobacillus* spp. have been shown to disrupt the cell membrane of fungi, leading to the leakage of cellular contents and, ultimately, cell death. These metabolites include hydrogen peroxide, which can induce oxidative stress in fungal cells, and bacteriocins such as nisin and pediocin, which can form pores in the cell membrane of fungi. Additionally, competition for nutrients is another antifungal mechanism of *Lactobacillus* spp. These strains can outcompete fungi for nutrients, thereby limiting their growth. This mechanism is particularly relevant in the gut, where Lactobacillus can compete with *Candida* species for nutrients [75]. Moreover, the metabolites of *Lactobacillus* spp. can enhance the host immune system, leading to the production of cytokines and other molecules that can inhibit the growth of fungi [76].

Another antifungal mechanism of probiotic bacterial strains is their efficacy to produce degrading enzymes such as chitinase and β-glucanase. These enzymes can degrade or weaken the fungal cell wall, making it more susceptible to other antifungal agents [77]. Interestingly, *Lactobacillus* spp. can secrete molecules that inhibit quorum sensing in fungi. Quorum sensing is a mechanism by which fungi communicate and coordinate their behavior, and inhibiting this mechanism can disrupt their ability to form biofilms and cause infections [78]. Based on the obtained data, it can be recommended to use the CFF of probiotic bacteria as safe, eco-friendly, and highly effective agents to reduce the deterioration of historical manuscripts caused by different fungal strains.

## 4. Conclusions

A historical manuscript that was in a state of high deterioration was investigated. The manuscript’s components, including the leather binding and paper sheets, showed signs of deterioration, such as stains caused by microbial contamination, erosion, weakness, brittleness, and other factors. The crystallinity index of the historical paper was lower than that of the new paper, which indicated that the historical paper suffered from deterioration which can be attributed to improper handling and environmental conditions. From these deteriorated samples, twelve fungal strains were isolated and identified as the following: two strains of *Cladosporium herbarum*, five strains of *Aspergillus fumigatus*, one strain of *A. ustus*, two strains of *A. flavus*, one strain of *A. niger*, and one strain of *Penicillium chrysogenum*. These fungal strains showed high enzymatic activity, which indicates their role in biodeterioration. The cell-free filtrate of the probiotic bacterial strains of *L. rhamnosus* contains various active metabolites that showed biocompatibility against two normal cell lines with high IC_50_ values. Different concentrations (300, 200, and 100 µg mL^−1^) of CFF showed antifungal activity against all fungal strains with percentages of 100%, whereas 50 µg mL^−1^ exhibited activity toward 66% of the total of fungal strains. Overall, the CFF derived from *L. rhamnosus* has the potential to prevent the growth of various fungal strains that are responsible for damaging historical papers. It is suggested that this CFF could be incorporated into preservation protocols.

## Figures and Tables

**Table 1:** Identification of different compounds present in the cell-free filtrate of *L. rhamnosus* using gas chromatography–mass spectrometry (GC-MS) based on different retention times (RT).

RT	Area %	Probability	Compound Name	Molecular Weight	Molecular Formula	Library
5.67	1.37	79.78	Acetic acid, butyl ester (CAS)	116	C_4_H_10_O_2_	Wiley9
5.79	1.87	43.80	1,3(R)Dihydroxybutane	90	C_4_H_10_O_2_	Wiley9
		8.66	Diethylene glycol	106	C_4_H_10_O_3_	nist_ms ms
		7.65	2Ethoxyethanol	90	C_4_H_10_O_2_	nist_ms ms
6.87	0.45	85.41	3,3Dimethoxy2butan one	132	C_6_H_12_O_3_	Wiley9
7.88	1.78	10.63	N-Acetyl glycine	117	C_4_H_7_NO_3_	nist_ms ms
		10.22	D-(-) Lactic acid	90	C_3_H_6_O_3_	
			L-(+)-Lactic acid			
9.32	3.59	70.99	Mono-Ethyl-malonate	132	C_5_H_8_O_4_	nist_ms ms
9.32	3.59	14.44	Oxalacetic acid	132	C_4_H_4_O_5_	
10.93	9.58	41.53	2Butanone, 3hydroxy-(CAS)	88	C_4_H_8_O_2_	Wiley9
		8.68	Methyl glyoxal	72	C_3_H_4_O_2_	mainlib
11.57	0.96	24.73	Propanoic acid, 2-hydroxy-(CAS)	90	C_3_H_6_O_3_	Wiley9
			(S)-2-Hydroxypropanoic acid			
			L-Lactic acid			mainlib
12.39	0.83	35.52	3-(3-chlorobutyl) isoquinolin-1(2H)-one	235	C_13_H_14_ClNO	Wiley9
		22.23	3-Pentanone, 1,5dimethoxy-(CAS)	146	C_7_H_14_O_3_	
			3-Pentanone, 1,5-dimethoxy-			mainlib
12.81	4.22	79.32	3-(t-Butoxy)-2-methylpropan-1-ol	144	C_8_H_16_O_2_	Wiley9
		5.49	2(S)-acetoxy-succinic anhydride	158	C_6_H_6_O_5_	
		5.28	(Z)-3-Tridecenal Diisopropyl Acetal	298	C_19_H_38_O_2_	
13.26	3.30	18.91	2-Butanone, 4-(acetyloxy)-(CAS)	130	C_6_H_10_O_3_	Wiley9
		16.71	2-Butanol, 3-Methyl, Acetate		C_7_H_14_O_2_	
		7.10	5-Hexen-2-one (CAS)	98	C_6_H_10_O	
15.20	0.66	16.61	Propanoic acid,2-ydroxy-, Methyl ester (CAS)	104	C_4_H_8_O_3_	Wiley9
		11.40	Acetic acid, methoxy-, ethyl ester (CAS)	118	C_5_H_10_O_3_	
		5.87	15-Crown-5	220	C_10_H_20_O_5_	mainlib
16.75	0.48	1.85	Phenyllacetic acid	136	C_8_H_8_O_2_	nist_ms ms
		1.35	2,6, Dimethyl-6-(phenyl methoxy)-2-heptene	232	C_16_H_24_O	Wiley9
			4-benzyloxy-3,5-dichloro-2-hydroxy-6-pentyl-benzoic acid	347	C_19_H_20_ClO_4_	
22.35	3.11	36.33	Benzene-propanoic acid, à-hydroxy-, methyl ester	180	C_10_H_12_O_3_	Wiley9
			Benzene-propanoic acid, à-hydroxy-, methyl ester (CAS)			
34.56	0.63	40.79	Hexadecanoic acid, methyl ester (CAS)	270	C_17_H_34_O_2_	Wiley9
37.93	3.58	10.03	cis-13-Octadecenoic acid, methyl ester	296	C_19_H_36_O_2_	mainlib
		8.86	9-Octadecenoic acid (Z)-, methyl ester			
		7.48	trans-13-Octadecenoic acid, methyl ester			
45.06	9.91	31.27	1,3-Benzenediamine, 2-methyl-5-nitro-(CAS)	167	C_7_H_9_N_3_O_2_	Wiley9
		11.42	4-(2,2-Dimethylpropyl)-2,2-dimethyl-3-methyl-netetrahydrofuran	182	C_12_H_22_O	
		10.53	7-Chloro-2-methylfuro [3,2-b] pyridine	167	C_8_H_6_ClNO	
45.14	2.63	27.62	9,9-Dimethyl-8, 10-diox-apentacyclo [5.3.0.0(2,5).0(3,5).0(3,6)] decane	166	C_10_H_14_O_2_	Wiley9
		12.58	4-(2-Propen-1-yloxy) benzene amine	149	C_9_H_11_NO	
		6.11	3-(5-’Formyl-2-furyl)-2-propenal	150	C_8_H_6_O_3_	
45.20	2.28	18.26	9,9-Dimethyl-8,10-diox apenta-cyclo [5.3.0.0(2,5).0(3,5).0(3,6)]decane	166	C_10_H_14_O_2_	Wiley9
		16.85	Methyl 1,3dihydro2Hisobenz ofuran-4-carboxylate	178	C_10_H_10_O_3_	
		12.57	Benzoic acid, 4-formyl-, ethyl ester (CAS)			
45.29	0.99	32.71	Pyrido [2,1-a] isoindolium Chloride	203	C_12_H_10_ClN	Wiley9
			Pyrido [2,1-a] isoindolium Bromide	247	C_12_H_10_BrN	
		6.78	2-Carboxy-4-methylbicyclo [2.2.2]oct-2-en-1-ol	182	C_10_H_14_O_3_	
45.49	47.78	81.24	8-Phenylacenaphtho [1,2-b] pyridine	279	C_21_H_13_N	Wiley9
		7.01	4-Amino5-cyano-6-(4′methoxyphenyl)-1-methyl-2,3-dihydropyrrolo [2,3-b] pyridine	280	C_16_H_16_N_4_O	
			10-Iodo-nido-7,8,9-phosphadicarbaborane		C_2_H_10_B_8_IP	

## Data Availability

The data presented in this study are available on request from the corresponding author.
